# Triglycerides and Sodium: Unmasking Pseudohyponatremia in a Clinical Case

**DOI:** 10.7759/cureus.74220

**Published:** 2024-11-22

**Authors:** Nusrat Hashem, Alina Abid, Siri Chandana Sola, Alexander Lewis

**Affiliations:** 1 Geriatrics, Leicester Royal Infirmary, Leicester, GBR; 2 Internal Medicine, University Hospitals of Leicester NHS Trust, Leicester, GBR; 3 Acute Medicine, University Hospitals of Leicester NHS Trust, Leicester, GBR; 4 Acute Medicine, Leicester Royal Infirmary, Leicester, GBR

**Keywords:** hyperproteinemia, hypertriglyceridemia, osmolarity, pseudohyponatremia, sodium

## Abstract

Sodium is one of the most important minerals in human blood. Sodium disorders, either in the form of hypernatremia or hyponatremia, have detrimental effects on the body; therefore, they warrant urgent attention. Hyponatremia occurs in various clinical scenarios; it can be further categorized as true hyponatremia and pseudohyponatremia. When blood gets high protein or fat contents, it factitiously lowers the sodium level, which is termed pseudohyponatremia. The management scheme for both true hyponatremia and pseudohyponatremia is different; hence, careful clinical co-relation is needed while dealing with such scenarios. We present a case of a 59-year-old gentleman referred by a general practitioner for severe hyponatremia. The patient demonstrated hyperglycemia on admission, hence started on variable rate insulin, and further investigations had been done to screen for metabolic syndrome. Routine investigations showed severe hypertriglyceridemia, leading to a diagnosis of pseudohyponatremia. The patient had been discharged with the dual anti-lipid-lowering and anti-hyperglycemic regimen. This case denotes the importance of understanding and differentiating pseudohyponatremia from true hyponatremia, which is essential to avoid mismanagement of sodium levels in clinical practice.

## Introduction

Electrolyte measurements are the most frequently ordered blood tests in modern clinical chemistry laboratories. The normal serum sodium concentration (Na) varies from 137 to 142 mmol/L. Hyponatremia represents the most common electrolyte disturbance seen in hospital practice [1]. Mild hyponatremia (serum sodium level between 130 and 134 mmol/L) occurs in 15-30% of hospitalized patients and 18% of nursing home individuals [1].

Hyponatremia can occur in hyperosmotic, isosmotic, and hypoosmotic plasma; hence, the measurement of plasma osmolarity is important in the assessment of hyponatremia. For example, in the presence of hyperlipidemia or hyperproteinemia, measured serum sodium can also be depressed without concomitant depression of serum osmolarity. This artifact of measurement is termed pseudohyponatremia in the context of physiologically normal sodium levels and pseudonormonatremia when the result masks physiologically increased sodium [2]. The water component gets diluted by non-aqueous materials like lipids and proteins in pseudohyponatremia. In conditions like hyperlipidemia and hyperproteinemia, abnormally high levels of these large molecules reconstitute the aqueous phase of plasma, thus leading to a reduced amount of serum electrolytes per unit volume of serum [3,4]. The clear concept of hypertriglyceridemia and pseudohyponatremia is essential for precise diagnosis and effectual treatment. This focuses the attention on how important it is to take lipid levels into account while assessing sodium discrepancy because they have a big impact on clinical assessment and management plans.

## Case presentation

A 59-year-old man presented to his general practitioner with a two-month history of fatigue, polyuria, and polydipsia. His general practitioner requested various blood tests, including lipid profile, HbA1c, urea, and electrolytes. Following these results, he was asked to return for another sample as there were concerns of spurious results as the patient was asymptomatic. Following the second set of results, he was asked to attend the accident and emergency department immediately. 

His blood tests within the emergency department showed a hyponatremia of 119 mmol/L (Table 1). The preceding blood tests requested by his general practitioner showed an HbA1c of 12.9 and triglycerides of 77.62 mmol/L. The rest of the hyponatremia screen was unremarkable. It was noted in the blood results that the sodium value should be interpreted with caution as the blood was severely lipemic. Full blood count and renal and liver function tests were unremarkable.

**Table 1:** Routine blood tests on admission. eGFR: estimated glomerular filtration rate, ALT: alanine aminotransferase.

Blood tests	18/10/2024	Normal values
HB	160 g/L	130-180 g/L
WBC	7 × 10^9^/L	4-11 × 10^9^/L
Platelet	182 × 10^9^/L	140-400 × 10^9^/L
Sodium	119 mmol/L	133-146 mmol/L
Potassium	4.3 mmol/L	3.5-5.3 mmol/L
Urea	7.5 mmol/L	2.5-7.8 mmol/L
Creatinine	107 umol/L	60-120 umol/L
eGFR	65 mL/min/1.73 m^2^	60-89 ml/min/1.73 m^2^
ALT	22 U/L	10-49 U/L
HBA1C	12.9%	4.0-5.9%
Calcium (adjusted)	2.40 mmol/L	2.12-2.51 mmol/L
Inorganic phosphate	1.27 mmol/L	0.80-1.50 mmol/L

On presentation to the accident and emergency department, the patient was found to be in non-acidotic diabetic ketosis and was started on variable rate insulin infusion. He was seen by the medical in-reach team working with the emergency department. 

He denied any other symptoms, no abdominal pain, no nausea or vomiting, and no chest pain on exertion or at rest. On examination, he was clinically euvolemic with no other remarkable findings. He had no signs of peripheral cholesterol deposits such as xanthoma, corneal arcus, or xanthelasma. 

He reported that his father had a history of high cholesterol and suffered a myocardial infarction at 67. He had a sibling who was well and had no diagnosis of hypercholesterolemia. Otherwise, the patient denied any other family history of high cholesterol. He denied drinking excessive alcohol (Table 2). 

**Table 2:** Patient's sodium and triglyceride levels during hospital stay.

	16/10/2024	18/10/2024	20/10/2024	Normal values
Na (mmol/L)	122	119	126	133-146 mmol/L
Triglyceride (mmol/L)	67.18	77.62	49.87	0.00-1.70 mmol/L
Cholesterol (mmol/L)	21.6	21.3	18.7	3.9-5.5 mmol/L

He had no history, which can suggest familial hypertriglyceridemia. We sent blood to monitor high-density lipoprotein (HDL) and low-density lipoprotein (LDL) levels. Unfortunately, the blood was unsuitable for analysis due to lipemia.

He was admitted to the acute medical unit, where his blood sugars stabilized. He was seen by the diabetic specialist nurses and started on metformin 1000 mg twice a day and Humulin I insulin 12 units in the morning and 10 units in the evening. Islet cell antibody, glutamic acid decarboxylase (GAD) antibody, IA2 antibody, and C-peptide were sent to diagnose whether it is type 1 or type 2 diabetes. All results came as negative for type 1 diabetes mellitus.

He had been commenced on atorvastatin 40 mg once a day and fenofibrate 160 mg once a day. He was discharged the following day with a planned virtual follow-up by the community diabetic nurses and in the clinic for further investigation of his raised triglycerides. Lifestyle advice had been given on discharge.

## Discussion

This case presents a patient with apparent hyponatremia in the setting of markedly elevated triglycerides (77 mmol/L), hyperglycemia, and ketonemia without acidosis. The case highlights the important clinical entity of pseudohyponatremia and demonstrates how severe hypertriglyceridemia can lead to spurious laboratory findings that might mislead clinical decision-making.

The key to understanding this case lies in the mechanism of pseudohyponatremia in the setting of severe hypertriglyceridemia. Plasma consists of approximately 93% water and 7% solutes. Electrolytes, like sodium ions, are almost completely dissociated in the water portion of plasma. To measure serum sodium levels, laboratories typically dilute the sample using a correction factor of 0.93. While these indirect methods provide accurate and reliable results under normal physiological conditions, when there is a significant increase in additional solutes, the water-to-solid ratio in plasma changes unpredictably, potentially causing inaccurate sodium ion measurements [5]. Not enough effect on other electrolytes. Direct ISE measurement, which measures sodium concentration in the plasma water phase only, provided a more accurate reflection of the patient's true sodium levels. When triglycerides are markedly elevated, as in our patient, they displace the aqueous portion of the blood where sodium actually resides, leading to an artificially low sodium measurement. This phenomenon is well-documented in cases where triglycerides exceed 10 mmol/L [5].

Our therapeutic approach focused on three critical aspects: treating hyperglycemia, severe hypertriglyceridemia, managing associated metabolic derangements, and careful monitoring. The presence of hyperglycemia and ketonemia required prompt attention through insulin therapy with regular blood glucose monitoring. We specifically avoided aggressive sodium correction, recognizing the spurious nature of low sodium measurements. Instead, we focused on regular assessment of clinical status and trending of laboratory parameters. The optimal method of insulin administration (whether intravenous or subcutaneous) for managing hypertriglyceridemia remains undetermined due to a lack of comparative research [6]. However, the therapeutic use of insulin in these cases has been validated by multiple studies, including Coskun et al.'s case series, which demonstrated insulin's effectiveness in treating hypertriglyceridemia, even in patients who had developed acute pancreatitis [7].

We initiated therapy with fibrates as first-line treatment, adding statins to optimize the overall lipid profile. Early consultation with the metabolic medicine team was arranged for specialized input and long-term management planning. Our management strategy emphasized treating the underlying causes rather than the apparent electrolyte abnormality, which proved effective in gradually improving the patient's metabolic status while avoiding iatrogenic complications.

Lifestyle modifications have been recommended to the patient to improve cardiovascular health. These changes, alongside medication, play a vital role in reducing cardiovascular risk and enhancing overall well-being. Aerobic exercise of moderate intensity with high frequency (about four hours per week) has been associated with the maintenance of improved cardiorespiratory fitness [8,9].

Patients with severe hypertriglyceridemia (triglyceride levels above 2000 mg per deciliter) associated with alcohol use should abstain [10]. The limited effect in patients with triglyceride levels below 500 mg per deciliter (5.6 mmol per liter) should not preclude moderate alcohol intake [11].

The finding of hypertriglyceridemia should prompt an investigation for other components of the metabolic syndrome. In particular, patients should be evaluated for fasting hyperglycemia, hypertension, abdominal obesity, and low HDL levels. Thyrotropin level, serum urea nitrogen, creatinine, and urinalysis should be obtained to assess thyroid and renal function [12].

We initiated therapy with fibrates as first-line treatment, adding statins to optimize the overall lipid profile. Early consultation with the metabolic medicine team was arranged for specialized input and long-term management planning. Our management strategy emphasized treating the underlying causes rather than the apparent electrolyte abnormality, which proved effective in gradually improving the patient's metabolic status while avoiding iatrogenic complications.

## Conclusions

In conclusion, this case report highlights the phenomenon of pseudohyponatremia secondary to elevated triglyceride levels, illustrating the importance of recognizing this condition in clinical practice. The patient's low serum sodium levels were misleading, as they did not reflect true hyponatremia but rather the interference of high triglycerides with standard laboratory measurements. In such a scenario, getting a full history with clinical examination is crucial when abnormal lab findings are noted. Assessing volume status clinically also gives guidance towards diagnosis. This case emphasizes the need for clinicians to consider alternative causes of hyponatremia, particularly in patients with hyperlipidemia, and to employ appropriate diagnostic strategies to avoid unnecessary interventions. Awareness of pseudohyponatremia can lead to more accurate interpretations of laboratory results and better patient management. Further research is warranted to explore the mechanisms behind this condition and to establish guidelines for its recognition and treatment.
